# Cortical Surface Spatial Analysis Reveals Altered Brain Functional Network Topology in T2DM With Mild Cognitive Impairment

**DOI:** 10.1002/brb3.70489

**Published:** 2025-04-21

**Authors:** YanJun fan, Jing Tian, XiaoMei Yu, Chen Yang, HuiYan Zhang, Jian Tan, YiWei Zhao, Jing Wei, Gang Huang, JiangPing Liu, LianPing Zhao

**Affiliations:** ^1^ The First Clinical Medical College of Gansu University of Chinese Medicine (Gansu Provincial Hospital) Lanzhou China; ^2^ Department of Radiology Gansu Provincial Hospital Lanzhou China; ^3^ Department of Radiology Ningxia Medical University General Hospital Yinchuan China; ^4^ NHC Key Laboratory of Diagnosis and Therapy of Gastrointestinal Tumor Gansu Provincial Hospital Lanzhou China

**Keywords:** brain functional network, graph theory, neuroimaging, T2DM

## Abstract

**Objective:**

Approximately 45.0% of patients who have type 2 diabetes mellitus (T2DM) exhibit mild cognitive impairment (MCI). However, the specific alternations in T2DM with MCI (T2DM‐MCI)‐related brain functional networks (BFN) remain unclear. Therefore, the present study aimed to investigate the alterations in the topological properties of BFN in T2DM patients with and without MCI, utilizing a cortical surface‐based graph theory analysis of resting‐state functional magnetic resonance imaging data.

**Methods:**

Neuropsychological performance and topological properties of BFNs were determined in 64 T2DM‐MCI patients, 58 T2DM patients without MCI (T2DM‐noMCI), and 78 healthy controls (HC). Moreover, we conducted the correlation and stepwise multiple linear regression analysis.

**Results:**

The T2DM‐MCI group showed increased global efficiency and decreased shortest path length compared to T2DM‐noMCI. In the left posterior cingulate, the T2DM‐MCI group exhibited higher nodal efficiency compared to the T2DM‐noMCI group. Additionally, both degree centrality and nodal efficiency in the T2DM‐noMCI group were significantly lower than in the HC. Degree centrality and nodal efficiency in the left basal ganglia were elevated in both T2DM groups. Alterations in these regions were related to cognitive function scores.

**Conclusion:**

The alterations in nodal properties of the left basal ganglia suggest that nodal attributes in this region may be involved in the neurophysiopathological mechanisms of brain injury in T2DM. Conversely, the alterations of nodal efficiency in the left posterior cingulate gyrus indicate its potential as a neuroimaging biomarker of cognitive impairment in T2DM patients.

## Graphical abstract

Altered nodal properties in the left basal ganglia may play a role in the neurophysiological mechanisms underlying brain injury in T2DM patients, while changes in the left posterior cingulate gyrus may serve as neuroimaging biomarkers for cognitive impairment.

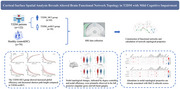

## 1 Introduction

Diabetes mellitus (DM) constitutes a prevalent chronic disease globally, with a steadily increasing incidence, accounting for 10.5% of individuals aged 20–79 years in 2021, affecting 536.6 million people (Sun et al. 2022, Aschner et al. 2021). Similarly, epidemiological surveys indicate that approximately 11.2% of adults in China have DM, with type 2 diabetes mellitus (T2DM) representing around 90% of cases (Chinese Diabetes Society 2021). Research indicates that T2DM can contribute to cognitive impairment (Chen et al. 2023), with mild cognitive impairment (MCI) accounting for approximately 45.0% among T2DM patients (You et al. 2021). In dementia etiology, DM is the largest possible variable risk factor (Diabetes Branch of Chinese Medical Association 2021, Ritchie et al. 2010). In addition, MCI is a preclinical stage of dementia and also a risk factor for its development. Research (Zhou et al. 2023) has uncovered that the three‐year conversion rate from MCI to dementia is 31%. Therefore, controlling the occurrence and progression of MCI may potentially delay the onset of dementia. However, the neurophysiopathological mechanisms by which T2DM leads to cognitive impairment remain poorly understood.

Magnetic resonance imaging (MRI) has been commonly utilized in studies of cognitive neuropsychological disorders due to its non‐invasiveness, lack of ionizing radiation, and high reproducibility. Nevertheless, most studies on T2DM have concentrated on structural brain changes or abnormalities in brain resting‐state functional activities (Qiu et al. 2024, Monereo‐Sánchez et al. 2023, Zhang et al. 2022), showing only a few specific or localized abnormal changes in brain regions. However, the human brain functions as a complex network, integrating and transmitting information across multiple regions and exhibiting important topological properties (Pervaiz et al. 2020, Ghaderi et al. 2021, Wang et al. 2013, Singh et al. 2023). Currently, graph theory‐based analytical methods have been employed to study brain networks in various neuropsychiatric disorders, but their application in T2DM research remains limited. For instance, a study of brain structural networks revealed reduced clustering coefficients and local efficiency (*E*_loc_) in T2DM patients without MCI (Huang et al. 2024). Conversely, a functional network study found significantly increased global efficiency (*E*_glob_) and *E*_loc_ in T2DM patients with MCI (Zhou et al. 2022). Another functional network study indicated that *E*_glob_ was similarly increased in T2DM patients without peripheral neuropathy (Xin et al. 2024). Therefore, the lack of consistency in previous research—owing to limited sample sizes, varying patient disease states, and differences in data analysis methods—has hindered a comprehensive understanding of the neurophysiological mechanisms underlying T2DM‐related cognitive impairment.

Functional parcellations have been reported to offer finer subdivisions than structural parcellations (Tian et al. 2020). Moreover, most previous studies on brain functional networks (BFN) have relied on three‐dimensional coordinates in volumetric space, which are susceptible to partial volume effects during cranial magnetic resonance data registration. In contrast, analyzing the cortical surface by reconstructing the cerebral cortex as a two‐dimensional cortical surface provides a superior representation of the brain's intricate curvilinear structure of the brain. Notably, the spatial orientation accuracy of traditional volume‐based methods is only 35% of that of cortical surface‐based analysis (Glasser et al. 2016, Coalson et al. 2018). Surface‐based approaches have been applied to studies of brain structure and resting‐state functional changes in conditions such as depression and T2DM (Liu et al. 2021, Tian et al. 2023), yielding new insights. However, this innovative technique has yet to be utilized in the study of BFN in T2DM.

Therefore, we aimed to assess the variances in the topological properties of BFNs in T2DM patients with (T2DM‐MCI) and without MCI (T2DM‐noMCI) via resting‐state functional MRI (rs‐fMRI) and cortical surface‐based graph theory analysis. Our findings may offer potential neuroimaging markers to detect early cognitive impairments in T2DM patients, offering them an objective imaging basis for early intervention, with the ultimate goal of enhancing their quality of life.

## 2 Methods

### 2.1 Participants

Our study recruited 178 T2DM patients from the Department of Endocrinology at Gansu Provincial Hospital between November 2017 and December 2023. Additionally, 104 healthy volunteers matched for sex, age, and education years were selected through advertisements to serve as healthy controls (HC group). The T2DM diagnosis was performed following the World Health Organization criteria in 1999 (Alberti and Zimmet 1998), which stipulate fasting blood glucose levels (BGLs) of ≥7.0 mmol/L or 2‐h postprandial BGLs of ≥11.1 mmol/L. Patients were subsequently categorized into two groups based on the Beijing version of the Montreal Cognitive Assessment Scale (MoCA): T2DM‐MCI group (MoCA  <  26) and T2DM‐noMCI group (MoCA  ≥  26) (Nasreddine et al. 2005, The Writing Group of Chinese Guidelines for the Diagnosis and Treatment of Dementia and Cognitive Impairment 2018). The HC group comprised individuals with random or fasting BGLs of <11.1 or <6.1 mmol/L, respectively, no DM history, and MoCA scores ≥26. Inclusion criteria included patients with right‐handedness, aged 18–65 years, and had at least six years of education. Exclusion criteria for this study were rigorously established to ensure participant safety and the integrity of the research findings. Individuals were excluded if they presented with organic lesions in the central nervous system hearing or visual impairments or had a history of cranial trauma or surgery. Furthermore, participants with psychiatric disorders or a family history of such conditions, as well as those struggling with alcohol dependence or substance abuse, were also excluded from the study. Additionally, contraindications to MRI disqualified certain individuals, as did the use of psychotropic medications within the preceding two months or the receipt of magnetic stimulation treatments in the prior 3 months. Pregnant or nursing women, as well as individuals currently taking oral contraceptives, were similarly excluded to minimize any potential risks. This study adhered to the Declaration of Helsinki and was authorized by the Medical Ethics Committee of Gansu Provincial Hospital (2017‐188, 2023–098), with all participants signing informed consent.

### 2.2 Clinical Data Collection and Neuropsychological Assessment

Comprehensive clinical data were meticulously collected from all participants, encompassing a range of parameters such as age, gender, body mass index (BMI), education years, T2DM duration for the patient cohort, and systolic (SBP) and diastolic blood pressure (DBP) measurements. To facilitate biochemical analyses, fasting venous blood samples were obtained from participants at 8:00 a.m. following more than a 10‐h fasting period. The collected samples were analyzed to assess a range of vital metrics, encompassing fasting BGLs, glycated hemoglobin, total cholesterol, triglycerides, high‐density lipoprotein and low‐density lipoprotein (LDL), C‐reactive protein, plasma cortisol, adrenocorticotropic hormone, triiodothyronine, thyroxine, and thyroid‐stimulating hormone. This extensive evaluation aimed to construct a detailed metabolic and hormonal profile of the participants, shedding light on their overall health status and potential risk factors associated with metabolic disorders. All participants were subjected to neuropsychological assessments via standardized instruments. The MoCA, known for its high sensitivity in detecting MCI (Jia et al. 2021, Bernier et al. 2023, Cogné et al. 2024, Bernardes et al. 2023), was employed to evaluate overall cognitive function. Moreover, we deployed the 24‐item Hamilton Depression Scale (HAMD‐24) alongside the Hamilton Anxiety Scale (HAMA) to assess the severity of depressive and anxiety symptoms, respectively. All assessments were completed within 1 h on the day of MRI scanning.

### 2.3 MRI Data Acquisition

MRI scans were conducted through a state‐of‐the‐art 3.0 T scanner (MAGNETOM Skyra, Siemens Healthineers, Germany) equipped with a 32‐channel phased‐array head coil at the Department of Radiology, Gansu Provincial Hospital. To ensure participant comfort and minimize exposure to the loud noises produced by the scanning process, individuals were positioned in a supine orientation and supplied with specialized headphones or earplugs designed specifically to dampen scanner noise. They were instructed to close their eyes, remain awake, and refrain from engaging in particular cognitive tasks throughout the scan. Initial MRI sequences included axial T1/2‐weighted images (T1/2WI) and T2‐fluid‐attenuated inversion recovery images to eliminate organic lesions and anatomical abnormalities. High‐resolution structural images and rs‐fMRI data were subsequently acquired to provide comprehensive insights into brain anatomy and connectivity. Structural imaging utilized a 3D T1‐weighted magnetization‐prepared rapid acquisition gradient echo (3D MPRAGE) sequence in the sagittal plane. The parameters included a repetition time (TR) of 2530 ms alongside an echo time (TE) of 2.35 ms, with a flip angle of 7°. The field of view (FOV) measured 256 × 256 mm^2^, and the image matrix was set at 256 × 256, voxel size of 1 × 1 × 1 mm^3^. Each acquisition produced 192 slices with a thickness of 1 mm, totally completed in an acquisition time of 5 min 23 s. For the rs‐fMRI data, we conducted a gradient‐echo echo‐planar imaging (GE‐EPI) sequence in the axial plane, with a TR of 2000 ms and a TE of 30 ms, using a flip angle of 90°. The FOV was set at 224 × 224 mm^2^, and the image matrix consisted of 64 × 64, with a slice thickness of 3.5 mm along with an inter‐slice gap of 0.875 mm. This sequence produced 33 slices across 420 volumes, ensuring robust and reliable data acquisition over an extended duration of 14 min and 8 s (Wehrheim et al. 2024, Birn et al. 2013, Anderson et al. 2011).

### 2.4 MRI Data Preprocessing

The MRI data preprocessing was carried out via DPABISurf V1.7 (Yan et al. 2021), built on MATLAB R2018b (https://www.mathworks.com/) as well as Statistical Parametric Mapping software (SPM12, http://www.fil.ion.ucl.ac.uk/spm12). Initially, we converted all MRI data from DICOM to NIfTI format and eliminated the first ten volumes to enable magnetization equilibrium and participant adaptation. The data were then organized relying on the Brain Imaging Data Structure format.

#### 2.4.1 Structural MRI Data Preprocessing

Structural MRI preprocessing involved several steps: (1) Intensity non‐uniformity correction: A correction was applied to the 3D T1WI to address inhomogeneities in the magnetic field (Tustison et al. 2010), establishing T1W references for subsequent processing; (2) Skull stripping: The skull was removed from the T1W reference images; (3) Tissue segmentation: Brain tissues were segmented into gray (GM) and white matter (WM), as well as cerebrospinal fluid (CSF) components (Zhang et al. 2001); (4) Surface reconstruction: Cortical surfaces were reconstructed using FreeSurfer version 6.0.1 (Dale et al. 1999); (5) Spatial normalization: Cortical surface‐based normalization was conducted from individual native space to the fsaverage template, while normalizing subcortical volumes to the Montreal Neurological Institute (MNI) standard space, ensuring comparability across subjects and enhancing analytical accuracy.

#### 2.4.2 Functional MRI Data Preprocessing

Functional MRI preprocessing included: (1) Co‐registration: rs‐fMRI reference images were subjected to co‐registration to the T1W reference using FreeSurfer's boundary‐based registration tool, bbregister (Greve and Fischl 2009); (2) Reference generation: We produced a reference volume and its skull‐stripped version employing fMRIPrep's custom methodology; (3) Slice timing correction: Temporal alignment of slices was corrected using 3dTshift (Parker and Razlighi 2019, Parker et al. 2017, Sladky et al. 2011); (4) Spatial normalization: Cortical rs‐fMRI time series were resampled to the fsaverage5 cortical template and further transformed into MNI space to produce preprocessed rs‐fMRI data aligned to standard coordinates; (5) Head motion correction: Estimating head motion parameters followed by calculating the framewise displacement (FD) for each participant (Power et al. 2014, Yang et al. 2021), excluding participants with mean FD  >  0.2  mm from further analysis (Yan et al. 2013); (6) Nuisance regression: Confounding signals were regressed out, including mean time series from WM and CSF (defined by FreeSurfer‐segmented masks), Friston‐24 motion parameters, and global signal (Hoeppli et al. 2023, Whitfield‐Gabrieli and Nieto‐Castanon 2012, Chai et al. 2012, Behzadi et al. 2007, Morfini et al. 2023); (7) Temporal filtering and smoothing: Band‐pass filtering (0.01–0.1 Hz) was conducted on the data, then spatial smoothing with a 6  mm full‐width at half‐maximum Gaussian kernel.

### 2.5 Construction of Functional Networks and Calculation of Network Topological Properties

The DPABINet V1.1 (Yan et al. 2024) network analysis module within the DPABI toolbox was utilized for constructing the functional network. Employing the Dosenback 160 template, the fMRI data were segmented into 142 and 18 regions of the cerebral cortex and the cerebellum, respectively. Subsequently, the average time series for each of the 142 regions of interest (ROIs) in the cerebral cortex was extracted, and the Pearson correlation between each ROI and all other ROIs was calculated, resulting in a 142 × 142 correlation matrix. GRETNA v2.0 (http://www.nitrc.org/projects/gretna) was utilized to compute both global and local network topological characteristics indicators. The global indicators encompass two network efficiency attributes and five small‐world attributes, including *E*_glob_, *E*_loc_, shortest path length (*L*p), clustering coefficient, standardized shortest path length (*γ*), standardized clustering coefficient (*λ*), and the small‐world property (*σ*). The local indicators primarily consist of the nodal clustering coefficient, nodal efficiency (*E*_nodal_), nodal *E*_loc_, degree centrality (DC), and nodal betweenness centrality. Herein, we established the sparsity threshold as 0.05–0.4, with a step size of 0.01.

### 2.6 Statistical Analysis

General clinical data, blood biochemical indicators, and neuropsychological scale assessment data were analyzed via SPSS (version 26, Inc., Chicago, IL, USA). One‐way ANOVA was deployed for continuous variables exhibiting a normal or approximately normal distribution, while the Kruskal–Wallis test was employed for continuous variables with a skewed distribution. The *χ*2 test was applied for categorical variables, such as sex. One‐way analysis of covariance (ANCOVA) was conducted using the GRETNA toolbox to compare network topological properties across three groups, controlling for gender, age, education years, and average FD as covariates. Topological indicators exhibiting statistically significant differences were extracted, and Bonferroni or Tamhane T2 post hoc comparisons were conducted in SPSS, with the selection based on the homogeneity of variances. For brain regions revealing significant inter‐group differences in nodal indicators, the parameter values of these nodal indicators were further extracted, and their correlations with the clinical parameters of patients with T2DM were calculated. Parameters that followed a normal or approximately normal distribution were analyzed using *Pearson* correlation, whereas non‐normally distributed parameters were assessed via *Spearman* correlation. Parameters demonstrating statistical significance were subjected to stepwise multiple linear regression to further validate the robustness of the correlations. *P* < 0.05 was deemed significant. Multiple comparison correction was carried out on the local topological properties indicators, as well as on the indicators under the global sparsity threshold, using the false discovery rate (FDR).

## 3 Results

### 3.1 Clinical Data and Demographics

Following exclusions due to left or double‐handedness (1 participant from the HC group) and excessive head motion during MRI scanning (3 from the T2DM‐MCI, 10 from the T2DM‐noMCI, and 2 from the HC groups), additional participants were removed to balance the groups for age, sex, and education level (13 from the T2DM‐MCI, 30 from the T2DM‐noMCI, and 22 from the HC groups). This led to a final sample of 201 participants: 64 in the T2DM‐MCI, 58 in the T2DM‐noMCI, and 79 in the HC groups. Table 1 summarizes demographic and clinical features. Unlike the T2DM‐noMCI group, LDL levels and the homeostatic model assessment of insulin resistance (HOMA2‐IR) were significantly reduced in the T2DM‐MCI group. In the T2DM‐noMCI group, DBP was significantly increased in comparison to the HC group. Both T2DM groups (MCI and noMCI) demonstrated significantly elevated HAMD‐24 and HAMA scores in contrast to the HC group. Additionally, the cognitive functions, as measured by the MoCA score, were significantly lower in the MCI group than in the T2DM‐noMCI and HC groups. Moreover, no statistical differences were observed for the remaining variables.

**TABLE 1:** Demographic data, clinical characteristic variables, and neuropsychological scales among T2DM‐MCI, T2DM‐noMCI, and HC groups.

	T2DM‐MCI (*n* = 64)	T2DM‐noMCI (*n* = 58)	HC (*n* = 79)	*t*/*F*/*Z*/*H*	*P*‐values
Sex (male/female)	48 M/16F	46 M/12F	53 M/26F	2.709	0.258
Age (years)	52.39 ± 7.63	51.57 ± 7.34	49.84 ± 8.13	2.041	0.133
Education (years)	12.47 ± 3.23	13.43 ± 2.12	12.60 ± 3.23	1.902	0.152
BMI (kg/m^2^)	24.24 ± 2.92	24.79 ± 2.43	23.63 ± 4.51	1.796	0.169
Disease duration (years)	7.00 (3.00, 10.00)	8.00 (5.00,10.00)		−0.963	0.336
HbA1c (%)	8.73 ± 2.39	8.79 ± 1.94		−0.140	0.146
FBG (mmol/L)	9.59 ± 4.07	10.10 ± 3.16		−0.760	0.185
HOMA2‐IR	1.03 (0.74,1.35)	1.27 (0.77,2.21)		−2.042	0.041*
TC (mmol/L)	4.36 ± 1.13	4.62 ± 1.06		−1.268	0.681
TG (mmol/L)	1.63 (1.18, 2.26)	2.02 (1.31, 3.08)		−1.085	0.278
HDL (mmol/L)	1.06 ± 0.22	1.05 ± 0.26		0.036	0.492
LDL (mmol/L)	2.27 ± 0.57	2.61 ± 0.87		−2.417	0.012*
CRP (mg/L)	0.85 (0.58, 1.95)	1.62 (0.93, 3.45)		−1.897	0.058
COR (nmol/L)	346.87 ± 80.10	345.90 ± 120.09		0.040	0.057
ACTH	38.55 (29.02, 50.19)	39.39 (26.14, 59.13)		−0.508	0.611
SBP (mmHg)	128.08 ± 19.78	128.95 ± ±14.71	121.69 ± 12.50	3.043	0.050
DBP (mmHg)	82.90 ± 12.76	85.37 ± 11.05 ^a^	79.71 ± 7.99	3.466	0.034*
BECK depression (score)	6.00 (3.00,10.50)	5.00 (3.00, 10.25)	5.00 (2.00, 9.00)	3.446	0.179
BECK anxiety (score)	5.00 (2.00,16.00)	4.00 (2.00, 9.25)	9.50 (1.00, 24.00)	4.055	0.132
HAMD‐24 (score)	8.00 (4.00,12.25)^a^	5.00 (2.00, 9.25)^a^	5.00 (0.00, 5.00)	31.075	<0.001*
HAMA (score)	3.50 (1.00,8.00)^a^	3.00 (1.00, 6.25)^a^	1.00 (0.00, 3.00)	18.489	<0.001*
MoCA (score)	22.73 ± 2.50^a,b^	27.55 ± 1.06	27.47 ± 1.34	164.716	<0.001*
Executive functions/Visuospatial abilities	3.03 ± 1.34^a,b^	4.22 ± 0.94	4.27 ± 0.86	27.364	<0.001*
Naming ability	2.86 ± 0.35^a,b^	3.00 ± 0.00	2.99 ± 0.12	8.015	<0.001*
Concentration	5.13 ± 0.92^a,b^	5.76 ± 0.47	5.70 ± 0.55	16.822	<0.001*
Language	2.14 ± 0.56^a^	2.31 ± 0.47^a^	2.63 ± 0.57	13.806	<0.001*
Verbal abstraction	1.52 ± 0.64^a,b^	1.86 ± 0.44	1.93 ± 0.27	14.001	<0.001*
Recall	2.00 (0.25,2.00)^a,b^	3.50 (3.00,4.00)	3.00 (2.00, 4.00)	68.758	<0.001*
Orientation	5.88 ± 0.38^a,b^	6.00 ± 0.00	6.00 ± 0.00	6.834	0.001*

Abbreviations: T2DM, type 2 diabetes mellitus; T2DM‐MCI, T2DM with mild cognitive impairment; T2DM‐noMCI, T2DM without mild cognitive impairment; HC: healthy controls; BMI, body mass index; SBP, systolic blood pressure; DBP, diastolic blood pressure; HbA1c, hemoglobin A1c; FBG, fasting blood‐glucose; HOMA2‐IR, homeostatic model assessment of insulin resistance; TC, total cholesterol; TG, triglyceride; HDL, high‐density lipoprotein; LDL, low‐density lipoprotein; CRP, creactive protein; COR, plasma cortisol; MoCA, Montreal cognitive assessment; HAMD‐24, Hamilton depression scale‐24; HAMA, Hamilton anxiety scale.

^a^ *P *< 0.05, compared with HC.

^b^ *P* < 0.05, compared with T2DM_noMCI.

*The difference was statistically significant.

### 3.2 Global Topological Properties of BFNs

**FIGURE 1:**
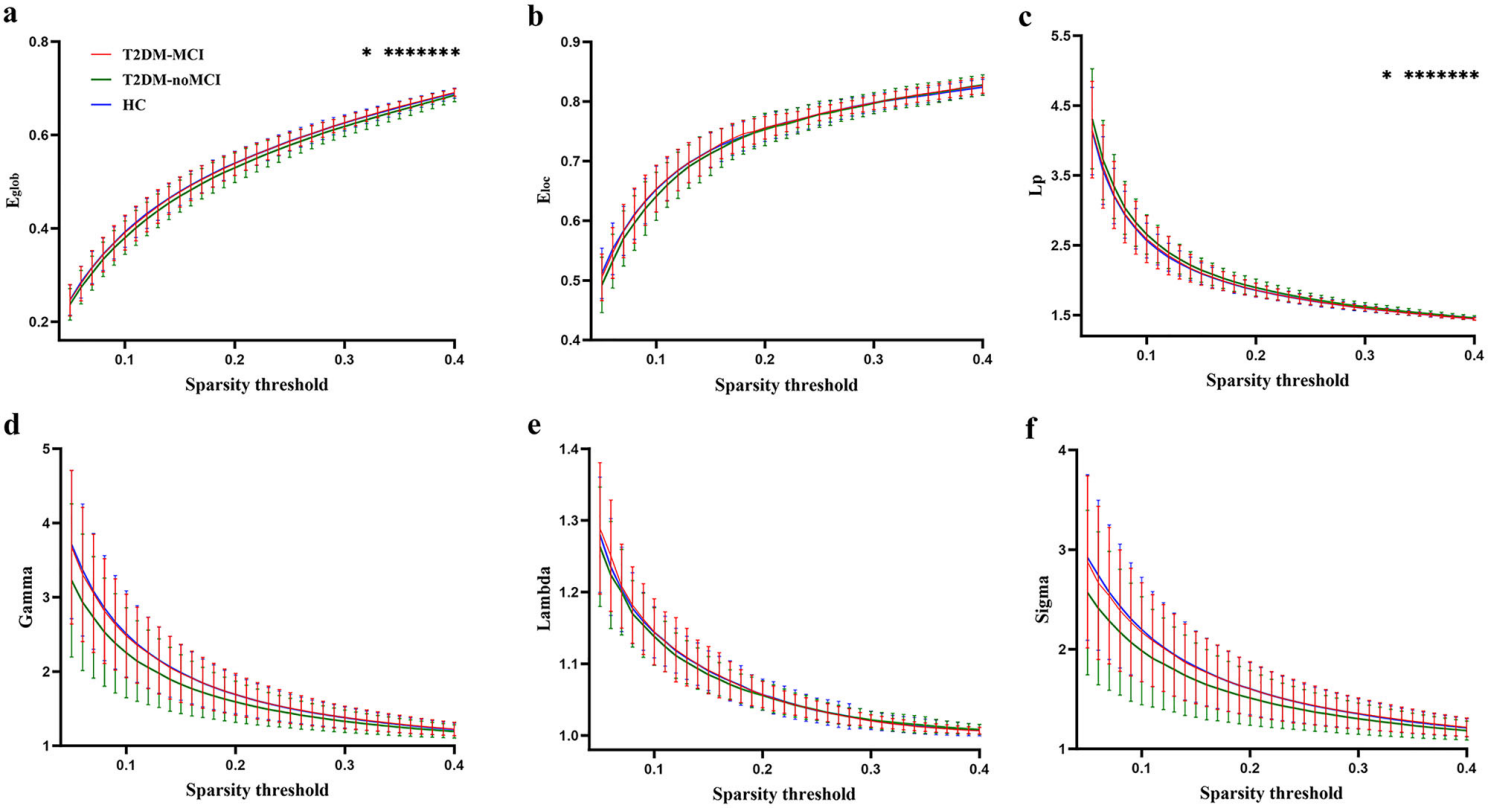
Comparison of global topological characteristics among T2DM‐MCI, T2DM‐noMCI, and HC. *P < 0.05: Intergroup significant differences. Key metrics include global (*E*_glob_) and local efficiency (*E*_loc_), shortest (Lp) and normalized shortest path length (*γ*), normalized clustering coefficient (*λ*), and small‐world properties (*σ* = *λ*/*γ*). Abbreviations: HC, healthy controls; T2DM, type 2 diabetes mellitus; T2DM‐MCI and T2DM‐noMCI, T2DM with and without mild cognitive impairment.

Within the 0.05–0.4 sparsity range (step size = 0.01), the three groups all demonstrated efficient small‐world topological properties (*γ* >1, *λ* ≈ 1, and *σ* = *γ*/*λ* > 1) (Figure 1). In comparison to the T2DM‐noMCI group, the T2DM‐MCI group demonstrated a significant escalation in *E*_glob_ and reduction in the Lp across a wide array of sparsity thresholds (*P* < 0.05, FDR correction, Figure 1). The other global topological properties did not statistically vary between the T2DM‐MCI and T2DM‐noMCI groups. Both T2DM groups displayed no significant difference in global attributes in contrast to the HC group. Although *E*_glob_ did not significantly differ between the T2DM‐noMCI and the HC groups, the T2DM‐noMCI group experienced a downward trend compared to the HC group.

### 3.3 Nodal Topological Properties of BFNs: Insights From Key Brain Regions

**FIGURE 2:**
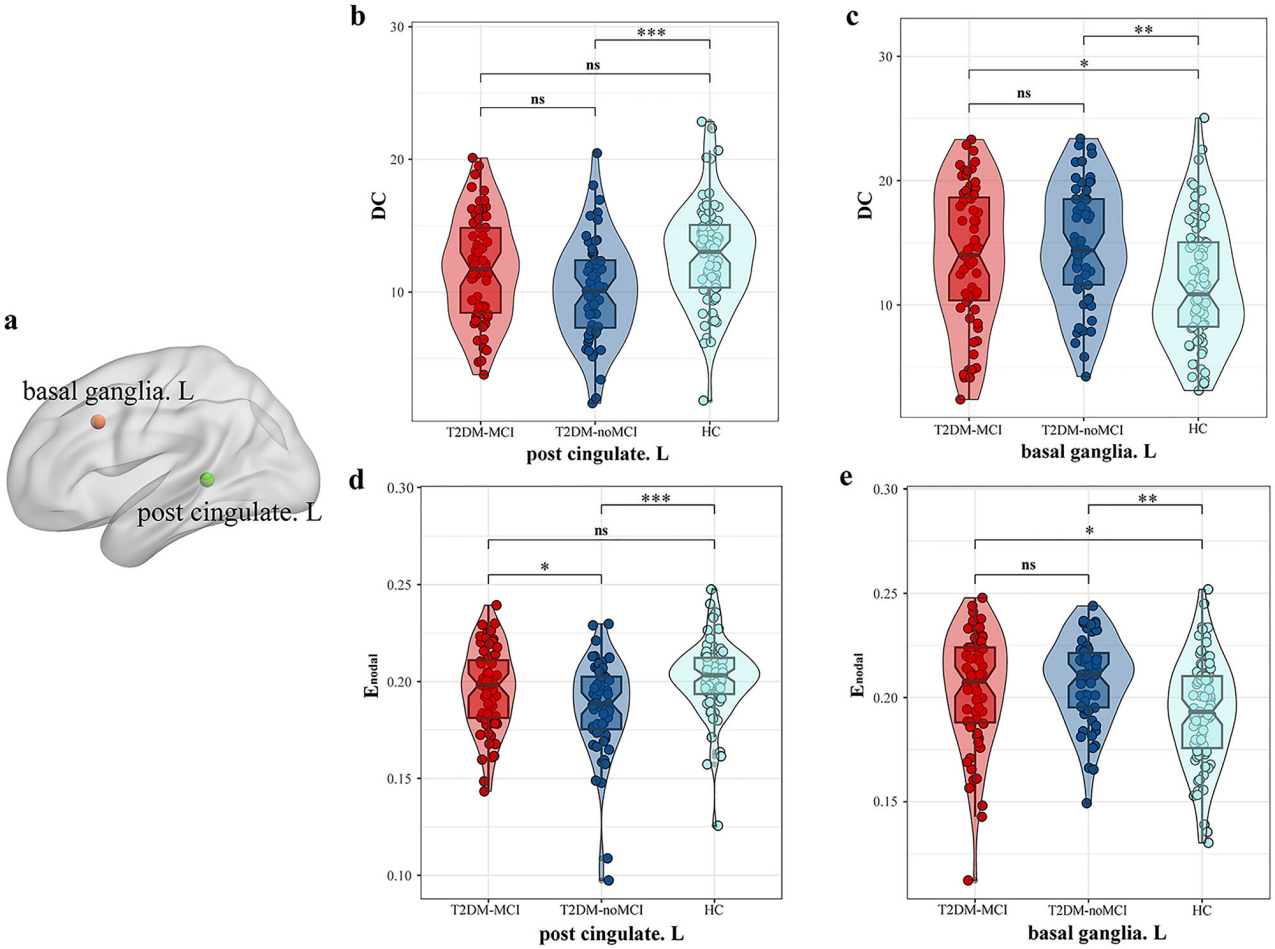
Nodal topological property comparisons among T2DM‐MCI, T2DM‐noMCI, and HC. Violin plots depict nodal characteristics, with asterisks indicating significant differences (*P* < 0.05). Abbreviations: DC, degree centrality; *E*_nodal_, nodal efficiency; HC, healthy controls; T2DM‐MCI and T2DM‐noMCI, T2DM with and without mild cognitive impairment; *P < 0.05; **P < 0.01; ***P < 0.001.

Changes in the nodal topological properties of T2DM patients were primarily recognized in the left posterior cingulate gyrus (PCG) and left basal ganglia (Table 2 and Figure 2). For DC, both the T2DM‐MCI and T2DM‐noMCI groups experienced a significant increase in the left basal ganglia more than the HC group, while the T2DM‐noMCI group elucidated a significant decrease in the left PCG. Although the DC in the left PCG of the T2DM‐MCI group displayed an upward trend, no statistical difference was found between the T2DM‐noMCI group. In terms of *E*_nodal_, both T2DM groups showcased a significant elevation in the left basal ganglia in contrast to the HC group, whereas the T2DM‐noMCI group revealed a significant decrease in the left PCG. Additionally, the T2DM‐MCI group manifested a significant rise in *E*_nodal_ in the left PCG in comparison to the T2DM‐noMCI group. Other nodal topological properties showed no statistically significant differences.

**TABLE 2:** Differential brain areas with significant changes in nodal topological properties among T2DM‐MCI group, T2DM‐noMCI group, and HC group.

	Labels	Brain regions	Coordinates MNI X Y Z			*F* values
DC						
	17	Post cingulate_L	−8	−41	3	9.138
	63	Basal ganglia _L	−6	17	34	9.628
*E_n_*_odal_						
	17	Post cingulate_L	−8	−41	3	8.869
	63	Basal ganglia _L	−6	17	34	8.688

Abbreviations: DC, degree centrality; *E*_nodal,_ nodal efficiency; Labels, the labels the Dosenbach 160 node atlas; T2DM‐MCI, T2DM with mild cognitive impairment; T2DM‐noMCI, T2DM without mild cognitive impairment; HC, healthy controls; L = left, R = right; MNI, Montreal neurological institute.

### 3.4 Correlations Between Clinical Variables and Topological Properties of Abnormal Brain Regions

**FIGURE 3:**
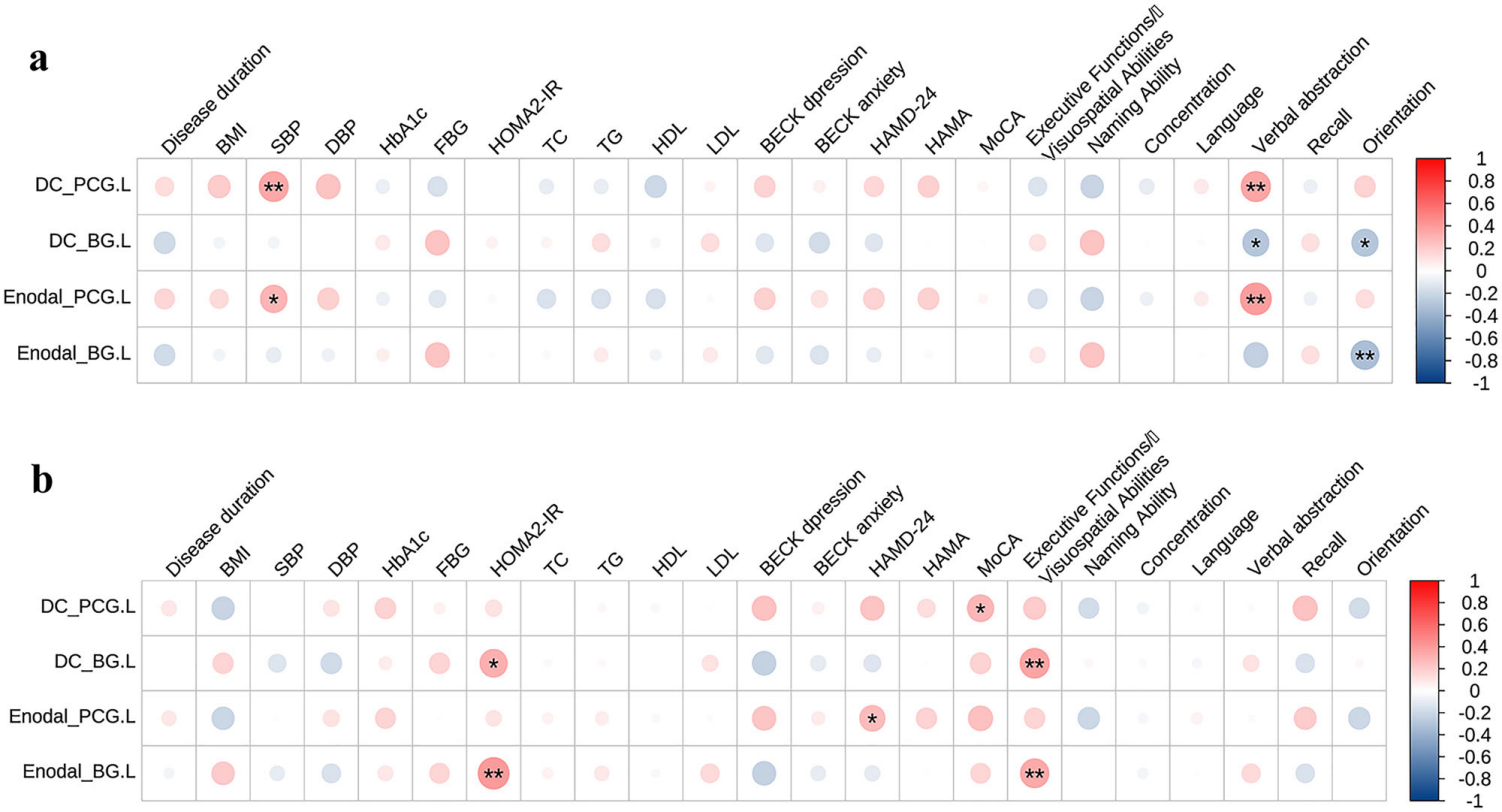
Correlations between nodal topological properties and clinical variables in the T2DM‐MCI and T2DM‐noMCI groups. Panel a shows T2DM‐MCI, while panel b shows T2DM‐noMCI. Notations: DC_PCG.L (degree centrality of the left posterior cingulate gyrus), DC_BG.L (degree centrality of the left basal ganglia), Enodal_PCG.L (nodal efficiency of the left posterior cingulate gyrus), Enodal_BG.L (nodal efficiency of the left basal ganglia). *P < 0.05; **P < 0.01.

Figure 3 illustrates the results of the correlation analysis regarding nodal topological properties indicators, clinical characteristic variables, and neuropsychological scales in patients who have T2DM accompanied by and not accompanied by MCI. Within the T2DM‐MCI group, the DC of the left PCG was positively linked to SBP (*r* = 0.351, *P* = 0.005) and verbal abstraction scores (*r* = 0.355, *P* = 0.004). Conversely, the DC of the left basal ganglia exhibited a negative connection to verbal abstraction (*r* = −0.280, *P* = 0.025) and orientation scores (*r* = −0.285, *P* = 0.022). Furthermore, the *E*_nodal_ of the left PCG exhibited a positive interplay with SBP (*r* = 0.294, *P* = 0.020) and verbal abstraction scores (*r* = 0.393, *P* = 0.001). The *E*_nodal_ of the left basal ganglia and orientation scores exhibited a negative association (*r* = −0.321, *P* = 0.010). In the T2DM‐noMCI group, the DC of the left PCG was positively related to the MoCA scores (*r* = 0.293, *P* = 0.026). The DC of the left basal ganglia showed a positive association with executive functions/visuospatial abilities (*r* = 0.361, *P* = 0.005) and HOMA2‐IR (*r* = 0.315, *P* = 0.020). Moreover, the *E*_nodal_ of the left PCG was positively linked to the HAMD‐24 scores (*r* = 0.270, *P* = 0.040). The *E*_nodal_ of the left basal ganglia was also positively correlated with executive functions/visuospatial abilities (*r* = 0.340, *P* = 0.009) and HOMA2‐IR (*r* = 0.403, *P* = 0.003). Notably, none of the above correlations survived after FDR correction.

### 3.5 Linear Regression Analysis

Linear regression analyses were carried out with nodal topological properties as dependent variables and clinical variables and neuropsychological scores as independent variables to identify significant independent variables. The T2DM‐MCI group (Table 3) displayed that DC of the left PCG was significantly connected to verbal abstraction scores (*β* = 0.325, *P*  =  0.007) and SBP (*β*  = 0.286, *P* =  0.017). The DC of the left basal ganglia was significantly related to verbal abstraction scores (*β*  =  −0.257, *P* =  0.038). *E*_nodal_ of the left PCG was significantly correlated to verbal abstraction scores (*β* =  0.330, *P*  =  0.007) and SBP (*β*  =  0.238, *P* = 0.049). *E*_nodal_ of the left basal ganglia was significantly interplayed with orientation scores (*β*  = −0.269, *P*  =  0.032). In the T2DM‐noMCI group, the following parameters exhibited stable effects on the dependent variables (Table 4): the DC of the left basal ganglia was significantly associated with the HOMA2‐IR (*β* = 0.331, *P* = 0.011) and executive functions/visuospatial abilities (*β* = 0.275, *P* = 0.033). Additionally, the *E*_nodal_ of the left basal ganglia was significantly correlated with HOMA2‐IR (*β* = 0.361, *P* = 0.006).

**TABLE 3:** Results of multiple linear regression stepwise analysis of the nodal properties in the T2DM‐MCI group.

		B	*β*	*t* values	*P* values	*F*	*R*	Adjusted *R* ^2^
DC_PCG.L								
	SBP	0.057	0.286	2.454	0.017	8.193	0.217	0.191
	Verbal abstraction	1.992	0.325	2.782	0.007			
DC_BG.L								
	Verbal abstraction	−2.210	−0.257	−2.116	0.038	4.144	0.120	0.091
*E*_nodal_ _PCG.L								
	SBP	0	0.238	2.009	0.049	6.976	0.191	0.164
	Verbal abstraction	0.011	0.330	2.785	0.007			
*E*_nodal_ _BG.L								
	Orientation	−0.020	−0.269	−2.197	0.032	4.826	0.072	0.057

Abbreviations: T2DM‐MCI, T2DM with mild cognitive impairment; SBP, systolic blood pressure; DC_PCG.L, the degree centrality of the left posterior cingulate gyrus; DC_BG.L, the degree centrality of the left basal ganglia; *E*_nodal_ _PCG.L, the nodal efficiency of the left posterior cingulate gyrus; *E*_nodal__BG.L, the nodal efficiency of the left basal ganglia; *B*, non‐standardized partial regression coefficient; ***β***, standardized partial regression coefficient; *R* ^2^, multiple correlation coefficient‐square.

**TABLE 4:** Results of multiple linear regression stepwise analysis of the nodal properties in the T2DM‐noMCI group.

		B	*β*	*t* values	*P* values	*F*	*R*	Adjusted *R* ^2^
DC_BG.L								
HOMA2‐IR		0.974	0.331	2.630	0.011	6.178	0.195	0.163
Executive/Visuospatial		1.441	0.275	2.189	0.033			
*E*_nodal_ _BG.L								
HOMA2‐IR		0.005	0.361	2.880	0.006	6.382	0.200	0.169

Abbreviations: T2DM‐noMCI, T2DM without mild cognitive impairment; DC_BG.L, the degree centrality of the left basal ganglia; HOMA2‐IR, homeostatic model assessment of insulin resistance; Executive/Visuospatial, Executive functions/Visuospatial abilities; *E*_nodal__BG.L, the nodal efficiency of the left basal ganglia; *B*, non‐standardized partial regression coefficient; *β*, standardized partial regression coefficient; *R* ^2^, multiple correlation coefficient‐square.

## 4 Discussion

This study, as far as we know, is the first to evaluate differences in the topological characteristics of BFNs among T2DM patients with and without MCI, as well as HCs, using cortical surface‐based graph theory analysis of rs‐fMRI data. Our principal findings are as follows: (1) T2DM patients exhibit significant alterations in the global and nodal attributes of BFN topology; (2) variations in global properties are primarily observed between T2DM‐MCI and T2DM‐noMCI groups, with the T2DM‐MCI group showing a significant escalation in *E*_glob_ and reduction in Lp across multiple sparsity thresholds; (3) nodal topological changes are mainly reflected in two indicators—DC and *E*_nodal_—and are predominantly located in the left PCG and left basal ganglia; (4) alterations in nodal topological properties are closely associated with MoCA subscale scores, particularly in verbal abstraction and orientation.

In this study, all three groups demonstrated high normalized clustering coefficient and low normalized shortest path length, which not only reflected the balance between the *E*_glob_ and *E*_loc_ of their brain networks (Yan et al. 2013) but also verified that the brain networks demonstrated small‐world topology, high connected hubs and modularity (Bullmore and Sporns 2009). The T2DM‐MCI group manifested a significant escalation in *E*_glob_ and a decrease in Lp across multiple sparsity ranges, unlike the T2DM‐noMCI group, aligning with previous findings (Zhou et al. 2022) and reinforcing the robustness of our results. While no statistically significant variances in global attributes were recognized when comparing both T2DM groups to HCs, a subtle increase in Lp was noted in the T2DM‐noMCI group, alongside a decreasing trend in *E*_glob_. Interestingly, this downward trend in *E*_glob_ contradicts the increasing trend reported by Zhou et al. (Zhou et al. 2022). Other studies (Xiong et al. 2022, Li et al. 2020) have also identified significant disturbances in the structural network at global and nodal levels in patients who have T2DM, particularly those with MCI, consistent with our observations in functional networks. Notably, previous research (Xin et al. 2024) has suggested that reductions in *E*_glob_ among T2DM patients with peripheral neuropathy may reflect a shift from compensatory mechanisms to decompensation, indicating that the BFN exhibits different trends at various disease stages. Our study differs from earlier investigations by featuring a relatively larger sample size and employing a cortical surface‐based analysis method, which offers greater accuracy than volume‐based approaches (Coalson et al. 2018). These methodological strengths enhance the reliability of our findings. Collectively, the results suggest that in the early stages of T2DM without MCI, there is a decrease in brain functional integration efficiency. As cognitive decline emerges with MCI, the brain may engage compensatory mechanisms, reflected by increased network efficiency, before eventually transitioning to decompensation with further disease progression and complications. Thus, changes in global topological properties in T2DM patients appear to be influenced by both the disease itself and alterations in cognitive function. Forthcoming studies using larger sample sizes, as well as more comprehensive subgroup analyses are essential for validating these speculations.

Herein, we observed that alterations in nodal attributes—specifically DC and *E*_nodal_—were predominantly located in the left PCG and the left basal ganglia. Growing evidence indicates that the cingulate gyrus, as one of the major connectivity hubs of cognition‐related brain networks, was strongly associated with all four domains of cognitive function comprising language, memory, attention/executive functions, and visuospatial processing (Metzler‐Baddeley et al. 2012, Kantarci et al. 2011). We found significant reductions in both DC and *E*_nodal_ in the left cingulate gyrus of the T2DM‐noMCI group compared to HCs, indicating a decrease in both the centrality of this region within the whole‐brain network and the efficiency of information transfer with adjacent nodes. These outcomes align with those reported by Xu et al. (Xu et al. 2019), who also identified the PCG as a key region exhibiting nodal property changes in T2DM patients, indicating its possibility as a neuroimaging biomarker for brain injury in this population. Although DC and *E*_nodal_ did not significantly differ between the T2DM‐MCI group and HCs, both metrics showed a decreasing trend. Correlation analyses and linear regression analysis further revealed that DC and *E*_nodal_ in the left PCG of the T2DM‐MCI group were connected to performance on the MoCA verbal abstraction subscale. These findings imply that alterations in the cingulate gyrus nodal properties are linked to cognitive impairment in T2DM patients and may underlie the neurophysiological mechanisms of cognitive decline. T2DM and hypertension are well‐established independent risk factors for cerebral small vessel disease (Sargurupremraj et al. 2020, Yang et al. 2023), a condition that is closely linked to cognitive decline (Teng et al. 2022). In this study, we identified a significant correlation between SBP and nodal properties in the left PCG among T2DM‐MCI patients, consistent with previous studies demonstrating the substantial impact of SBP on the T2DM brain (de Havenon et al. 2019). Although nearly all participants’ blood pressure was within the reference range, the T2DM group exhibited a trend toward higher SBP compared to the HC group. This observation suggests that elevated SBP may play a potential role in the pathophysiological mechanisms underlying T2DM‐related brain alterations. Our findings underscore the imperative for further investigation into the intricate interplay between SBP and T2DM, as well as the underlying mechanisms contributing to cognitive dysfunction. Intriguingly, we also recognized a significant rise in *E*_nodal_ in the left PCG of the T2DM‐MCI group in comparison to the T2DM‐noMCI group, with DC showing an upward trend. This pattern mirrors the findings by Zhou et al. (Zhou et al. 2022), who reported increased DC and E_nodal_ in multiple brain regions among T2DM‐MCI patients relative to those without MCI. Based on these observations, we hypothesize that DC and E_nodal_ in the left PCG decrease during the early stages of T2DM but may experience compensatory increases to mitigate cognitive decline as MCI develops. Therefore, alterations in the nodal topological properties of the left PCG may be modulated by both T2DM progression and cognitive deterioration rather than by a single factor.

Compared to HCs, T2DM patients—with or without MCI—highlighted significant increases in DC and *E*_nodal_ in the left basal ganglia. In the structural brain networks of T2DM patients, the basal ganglia have been identified as key regions with abnormal nodal parameters (Zhang et al. 2019). Functionally, the basal ganglia are divided into sensorimotor, associative, and limbic, responsible for processing motor, cognitive, and emotional or motivational information, respectively (Yelnik 2002). Choi et al. (Choi et al. 2023) reported a linear decrease in GM volume within the basal ganglia during T2DM, a reduction often accompanied by neuronal loss (Pakkenberg and Gundersen 1997, Stark et al. 2007, Otsuka et al. 2022, Preziosa et al. 2023, Gingele et al. 2024, Mcmahan et al. 2023, Gaus et al. 2023), which can impede information transfer. Thus, the observed increases in DC and *E*_nodal_ may represent compensatory mechanisms whereby T2DM patients enhance information transfer efficiency in response to neuronal loss. Our study also found that *E*_nodal_ in the left basal ganglia of the T2DM‐MCI group was negatively correlated with orientation scores, further suggesting that increased nodal properties in this region participate in compensatory processes following brain injury in T2DM patients. We also found that in the T2DM‐noMCI group, both the DC and *E*_nodal_ of the left basal ganglia were positively correlated with the HOMA2‐IR, suggesting that under the influence of more severe glucose metabolism disturbances, the associated neural compensatory mechanisms may become more active. Although this aligns with our previous conclusions (Yang et al. 2024), it should be noted that these results are derived from correlation analyses and thus require further validation in future studies. Since no significant variations were detected between T2DM‐MCI and T2DM‐noMCI groups, the relation between cognitive function changes and nodal properties in the basal ganglia appears limited.

This study innovatively utilized cortical surface‐based graph theory analysis to elucidate changes in BFN topology among patients who have T2DM accompanied by and not accompanied by MCI. However, multiple limitations warrant consideration. First, due to the cross‐sectional study design, the causal interplay between the alterations in topological properties and the progression of T2DM and changes in cognitive‐emotional function cannot be determined. Second, individual differences among T2DM patients, including the types and doses of hypoglycemic agents used, may influence nervous system function and were not controlled. Moreover, the absence of a control group with MCI but without diabetes makes it challenging to determine precisely whether changes in brain network parameters are primarily attributable to diabetes or cognitive impairment. Finally, the exclusion of T2DM patients with prediabetes and complications limits our ability to characterize topological property changes across different disease stages. Future studies should employ larger sample sizes and refined subgroup analyses to address these limitations.

## 5 Conclusions

In conclusion, T2DM patients exhibit abnormal topological properties in BFNs, irrespective of MCI presence. Specifically, altered nodal properties in the left basal ganglia may have a function in the neurophysiological mechanisms underlying brain injury in T2DM patients, while changes in the left PCG may serve as neuroimaging biomarkers for cognitive impairment. Our study offers novel perspectives into the neuropathophysiological mechanisms behind cognitive decline in T2DM patients besides providing objective imaging evidence to inform new diagnostic and therapeutic strategy development aimed at ameliorating cognitive abnormalities in this population.

## Author Contributions

YanJun Fan recruited participants, collected clinical data, MRI data, and neurophysiological assessments, analyzed data, and drafted and wrote the manuscripts. Jing Tian, XiaoMei Yu, Chen Yang, HuiYan Zhang, Jian Tan, YiWei Zhao, and Jing Wei recruited participants and collected clinical data, MRI data, and neurophysiological assessments. Gang Huang and JiangPing Liu coordinated magnetic resonance scans and provided technical and material support. LianPing Zhao is the principal architect of this work, having conceived, designed, and revised the manuscript. As the guarantor, Zhao holds complete access to all study data and assumes full responsibility for both data integrity and analysis accuracy. All have reviewed, read, and approved the final manuscript.

### Ethics Statement

The Medical Ethics Committee of Gansu Provincial Hospital (2017‐188, 2023–098), with all participants signing informed consent.

### Peer Review

The peer review history for this article is available at https://publons.com/publon/10.1002/brb3.70489

## Data Availability

Generated/analyzed datasets contain sensitive personal information from patients and are not publicly available. However, these datasets can be accessed upon request.
